# Spontaneous Amputation in a New-born Child

**Published:** 1847-07

**Authors:** 


*Spontaneous Amputation in a new-born Child.*—M. Paul Dubois presented to the Academy of Medicine a child, two days old, which presented remarkable and rare congenital lesions. Immediately after its birth, it was perceived that the middle and ring fingers of the left hand were reduced to the first phalanges; the free extremities of the latter were rounded, and covered over with skin, except at a small part, which still presented a wound, and showed the removal of the distal phalanges to have been recent. From alongside these small wounds, arose a slender but resisting filiform prolongation, larger than the wanting phalanges would have been, otherwise it might be considered as the remains of them.

A similar lesion existed in the second and third toes of the left, and also of the right foot. The last phalanges were wanting, and stumps replaced them, presenting central wounds and filiform appendages, as in the hand.

The left leg presented, a little above the malleoli, an obvious constriction, circular and straight, as though it had been produced by a ligature, but no vestige of such a thing was to be found. The great toe of the right foot offered, on a level with its first phalanx, a similar constriction. This alteration, and the removal of the toes, seemed to constitute two stages of the malady. Lastly, the right leg also presented a circular depression, having the same characters, and occupying the same position, as the constriction on the left leg, but much less marked. At the time of birth, no trace of inflammation existed around the mutilated parts; but since, and under the influence of the new conditions of external existence, a true inflammatory state had been set up.

The umbilical cord was but half its usual length; the membranes enclosing the child seemed to be constituted only by the chorion; at least, the amnion could not be distinguished. The placenta offered nothing remarkable. Setting aside the mutilations described, the child was well formed and fully developed.

The mother was not taken into the hospital until after the membranes had burst, and it was impossible to discover any trace of the deficient members.—*Ibid.*

